# Draft Genome Sequence of *Ralstonia* sp. Strain SET104, Isolated from Root Nodules of *Aeschynomene indica*

**DOI:** 10.1128/MRA.01441-18

**Published:** 2019-01-10

**Authors:** Aiko Tanaka, Takamasa Suzuki, Kazuma Uesaka, Shingo Hata

**Affiliations:** a Graduate School of Bioagricultural Sciences, Nagoya University, Nagoya, Japan; b Faculty of Agriculture, Ryukoku University, Otsu, Shiga, Japan; c College of Bioscience and Biotechnology, Chubu University, Kasugai, Aichi, Japan; d Center for Gene Research, Nagoya University, Nagoya, Japan; Georgia Institute of Technology

## Abstract

Here, we report a draft genome sequence of *Ralstonia* sp. strain SET104, isolated from the root nodules of Aeschynomene indica. The assembled draft genome size was 4,796,748 bp, containing a predicted total of 4,464 protein-encoding sequences. SET104 appears to be a novel species of the genus *Ralstonia*.

## Abstract (precis)

Here, we report a draft genome sequence of *Ralstonia* sp. strain SET104, isolated from the root nodules of Aeschynomene indica.

## ANNOUNCEMENT

Aeschynomene indica is known to form nitrogen-fixing root and stem nodules in association with bacteria in the family *Bradyrhizobiaceae* (1, 2) through intercellular infection (1, 3) that is independent of nodulation (Nod) factors (4, 5). Since this process is thought to be the basis of bacterial infection in leguminous plants (3, 6), an understanding of the mechanism involved will elucidate how legumes evolved these symbiotic relationships.

We collected A. indica seeds from wild plants growing in Sasayama, Japan, and confirmed their identity by sequencing the nuclear ribosomal DNA internal transcribed spacer (ITS)/5.8S region and the chloroplast DNA *trnL* intron (7). We germinated the seeds and grew the resulting seedlings in volcanic ash soil supplemented with nitrogen-free medium (8) and water in a paddy field in Sasayama. After 2 months, roots with nodules were harvested, surface sterilized, crushed, and streaked on arabinose-gluconate agar plates (9) to isolate the bacteria in the nodules. We then reinoculated the A. indica seedlings with the isolated bacteria under sterilized conditions. One of the isolated strains, named SET104, was identified as a *Ralstonia* sp. based on partial sequencing of the 16S rRNA gene. Since there have been no previous reports of nitrogen fixation by a *Ralstonia* sp., we focused on producing a draft genome for SET104.

Genomic DNA was extracted from strain SET104 cells using a genomic DNA purification kit (Promega, USA) following the manufacturer’s protocol. DNA libraries were prepared using a TruSeq DNA sample prep kit (Illumina, USA) and sequenced with the NextSeq 500 sequencer (Illumina, USA). Paired-end sequencing generated a total of 28,752,817 reads with an average read length of 100 bp. The reads were quality trimmed using the FASTQ preprocessing program fastp (10). *De novo* assembly was then performed using the SPAdes genome assembler version 3.12 (11) with two options (“–k auto” and “–careful”), which yielded 58 contigs with an *N*_50_ value of 287,261 bp and 59-fold mean coverage. The resulting draft genome of strain SET104 consisted of 4,796,748 bases with a GC content of 63.3%. Annotation of the contigs was performed using the DFAST version 1.0.8 pipeline (12), which identified 4,464 putative coding sequences.

Genome annotation indicated an absence of the *nifH* gene, which encodes nitrogenase iron protein, and other genes that are required for nitrogen fixation. Furthermore, although the *nodI* and *nodJ* genes were found to be present, the canonical nodulation genes (*nodA*, *nodB*, and *nodC*) were not detected, indicating that strain SET104 does not produce Nod factors.

Application of the genome-based distance matrix calculator (http://enve-omics.ce.gatech.edu/g-matrix/index) revealed that SET104 had average nucleotide identity (ANI) and average amino acid identity (AAI) values of 88% and 91%, respectively, in relation to the closest species, Ralstonia pickettii. Since genomes with an ANI of >95% and/or an AAI of >95% are generally considered to have originated from the same species (13–15), we believe that SET104 represents a novel species of *Ralstonia*.

### Data availability.

The genome sequence of *Ralstonia* sp. strain SET104 was deposited in DDBJ/EMBL/GenBank under accession numbers BHVX01000001 to BHVX01000058. The raw sequencing reads have been submitted to the Sequence Read Archive (SRA) under the accession number DRA007473.

## References

[1] Bonaldi K, Gargani D, Prin Y, Fardoux J, Gully D, Nouwen N, Goormachtig S, Giraud E 2011 Nodulation of *Aeschynomene afraspera* and *A. indica* by photosynthetic *Bradyrhizobium* sp. strain ORS285: the Nod-dependent versus the Nod-independent symbiotic interaction. Mol Plant Mocrobe Interact 24:1359–1371. doi:10.1094/MPMI-04-11-0093. 21995799

[2] Okubo T, Fukushima S, Itakura M, Oshima K, Longtonglang A, Teaumroong N, Mitsui H, Hattori M, Hattori R, Hattori T, Minamisawa K 2013 Genome analysis suggests that the soil oligotrophic bacterium *Agromonas oligotrophica* (*Bradyrhizobium oligotrophicum*) is a nitrogen-fixing symbiont of *Aeschynomene indica*. Appl Environ Microbiol 79:2542–2551. doi:10.1128/AEM.00009-13. 23396330 PMC3623176

[3] Madsen LH, Tirichine L, Jurkiewicz A, Sullivan JT, Heckmann AB, Bek AS, Ronson CW, James EK, Stougaard J 2010 The molecular network governing nodule organogenesis and infection in the model legume *Lotus japonicus*. Nat Commun 1:10. doi:10.1038/ncomms1009. 20975672 PMC2892300

[4] Giraud E, Moulin L, Vallenet D, Barbe V, Cytryn E, Avarre J-C, Jaubert M, Simon D, Cartieaux F, Prin Y, Bena G, Hannibal L, Fardoux J, Kojadinovic M, Vuillet L, Lajus A, Cruveiller S, Rouy Z, Mangenot S, Segurens B, Dossat C, Franck WL, Chang W-S, Saunders E, Bruce D, Richardson P, Normand P, Dreyfus B, Pignol D, Stacey G, Emerich D, Verméglio A, Médigue C, Sadowsky M 2007 Legumes symbioses: absence of Nod genes in photosynthetic bradyrhizobia. Science 316:1307–1312. doi:10.1126/science.1139548. 17540897

[5] Miché L, Moulin L, Chaintreuil C, Contreras-Jimenez JL, Munive-Hernández J-A, Villegas-Hernandez M, Crozier F, Béna G 2010 Diversity analyses of *Aeschynomene* symbionts in tropical Africa and Central America reveal that *nod*-independent stem nodulation is not restricted to photosynthetic bradyrhizobia. Environ Microbiol 12:2152–2164. 10.1111/j.1462-2920.2009.02090.x. 21966910

[6] Sprent JI 2007 Evolving ideas of legume evolution and diversity: a taxonomic perspective on the occurrence of nodulation. New Phytol 174:11–25. doi:10.1111/j.1469-8137.2007.02015.x. 17335493

[7] Chaintreuil C, Arrighi J-F, Giraud E, Miché L, Moulin L, Dreyfus B, Munive-Hernández J-A, Villegas-Hernandez Mdel C, Béna G 2013 Evolution of symbiosis in the legume genus *Aeschynomene*. New Phytol 200:1247–1259. doi:10.1111/nph.12424. 23879229

[8] Broughton WJ, Dilworth MJ 1971 Control of leghaemoglobin synthesis in snake beans. Biochem J 125:1075–1080. doi:10.1042/bj1251075. 5144223 PMC1178271

[9] Sadowsky MJ, Tully RE, Cregan PB, Keyser HH 1987 Genetic diversity in *Bradyrhizobium japonicum* serogroup 123 and its relation to genotype-specific nodulation of soybean. Appl Environ Microbiol 53:2624–2630. 16347481 10.1128/aem.53.11.2624-2630.1987 PMC204163

[10] Chen S, Zhou Y, Chen Y, Gu J 2018 fastp: an ultra-fast all-in-one FASTQ preprocessor. Bioinformatics 34:i884–i890. doi:10.1093/bioinformatics/bty560. 30423086 PMC6129281

[11] Bankevich A, Nurk S, Antipov D, Gurevich AA, Dvorkin M, Kulikov AS, Lesin VM, Nikolenko SI, Pham S, Prjibelski AD, Pyshkin AV, Sirotkin AV, Vyahhi N, Tesler G, Alekseyev MA, Pevzner PA 2012 SPAdes: a new genome assembly algorithm and its applications to single-cell sequencing. J Comput Biol 19:455–477. doi:10.1089/cmb.2012.0021. 22506599 PMC3342519

[12] Tanizawa Y, Fujisawa T, Nakamura Y 2018 DFAST: a flexible prokaryotic genome annotation pipeline for faster genome publication. Bioinformatics 34:1037–1039. doi:10.1093/bioinformatics/btx713. 29106469 PMC5860143

[13] Goris J, Konstantinidis KT, Klappenbach JA, Coenye T, Vandamme P, Tiedje JM 2007 DNA-DNA hybridization values and their relationship to whole-genome sequence similarities. Int J Syst Evol Microbiol 57:81–91. doi:10.1099/ijs.0.64483-0. 17220447

[14] Konstantinidis KT, Tiedje JM 2005 Towards a genome-based taxonomy for prokaryotes. J Bacteriol 187:6258–6264. doi:10.1128/JB.187.18.6258-6264.2005. 16159757 PMC1236649

[15] Thompson CC, Chimetto L, Edwards RA, Swings J, Stackebrandt E, Thompson FL 2013 Microbial genomic taxonomy. BMC Genomics 14:913. doi:10.1186/1471-2164-14-913. 24365132 PMC3879651

